# GLUT2 proteins and PPARγ transcripts levels are increased in liver of ovariectomized rats: reversal effects of resistance training

**DOI:** 10.20463/jenb.2016.06.20.2.7

**Published:** 2016-06-30

**Authors:** Luciane M. Tomaz, Marina R. Barbosa, Zahra Farahnak, Cristiani G. Lagoeiro, Natalia S.S Magosso, Jean-Marc Lavoie, Sérgio E. A. Perez

**Affiliations:** 1 Department of Physiological Sciences, Federal University of São Carlos, São Carlos, SP Brazil; 2 Department of Education and Health, Federal University of Sergipe, Lagarto, SE Brazil; 3 Department of Kinesiology, Université de Montréal, Montréal, QC Canada

**Keywords:** Liver fat, PEPCK, Hepatic glycogen, Exercise and Glucose

## Abstract

**[Purpose]:**

This study investigated the effects of ovariectomy (Ovx) and 12 weeks of resistance training (RT) on gene expression of GLUT2, the main glucose transporter in the liver, and on PPARγ, a transcription factor known to target GLUT2 expression.

**[Methods]:**

Forty Holtzman rats were divided into 5 groups: Sham-sedentary (Sed), Sham- RT, Ovx-Sed, Ovx-RT, and Ovx-Sed with hormone replacement (E2). The RT protocol consisted of sessions held every 72 h for 12 weeks, during which the animals performed 4 to 9 vertical climbs (1.1 m) at 2 min intervals with progressively heavier weights (30 g after the fourth climb) tied to the tail. The E2 silastic capsule was inserted into the rats’ backs 48 hours before the first RT session.

**[Results]:**

In addition to liver fat, GLUT2 protein levels and PPARγ transcripts were increased (*P* < 0.05) in Ovx compared to Sham-Sed animals, suggesting increased hepatic glucose uptake under estrogen deficient conditions. RT and E2 in Ovx rats decreased liver fat accumulation as well as GLUT2 and PPARγ gene expression to the level of Sham-Sed animals.

**[Conclusion]:**

The results of this study suggest that liver GLUT2 as well as PPARγ expression in Ovx rats are accompanied by increased fat accumulation and glucose uptake, thus providing a substrate for increased *de novo* lipogenesis. RT appears to be an appropriate exercise model to circumvent these effects.

## INTRODUCTION

Menopause, as well as estrogen withdrawal in rats through ovariectomy or other animal models of menopause, is associated with several metabolic changes that include increased liver fat deposition^1-3^. Liver fat accumulation is recognized as the hepatic component of metabolic syndrome^4^.

The specific origin of the lipids that accumulate in rat liver under estrogen withdrawal remains unclear. Measurements of molecular markers in different studies suggest a reduction in lipid oxidation and in the production of very low density lipoprotein triglycerides (VLDL-TG)5,6 and an increase in *de novo* lipogenesis^7,8^. Several molecular markers of *de novo* lipogenesis in the liver, including the transcription factor sterol regulatory element-binding protein 1c (SREBP-1c) and its downstream enzyme stearoyl-CoA desaturase-1 (SCD-1), have been reported to be greatly increased in ovariectomized rats^7,8^. If, indeed, *de novo* lipogenesis is increased in the livers of Ovx rats, then extra glucose molecules must be available as substrates for lipid synthesis. Therefore, we postulated that Ovx-induced liver fat accumulation would also result in increased hepatic glucose uptake through increased expression of glucose transporters. The first purpose of the present study was to test the hypothesis that hepatic gene expression of GLUT2, the main glucose transporter in hepatocytes of rodents and humans^9^, is increased in Ovx rats. In addition, we also targeted the transcription factor PPARγ. Gene expression of PPARγ is low in liver tissue^10^. Nevertheless, there is some evidence that liver GLUT2 is a direct target of PPARγ, thus contributing to glucose transport into the liver^11^.

There is accumulating evidence that endurance exercise training overcomes several of the metabolic effects of ovariectomy in rats. For instance, Ovx animals can benefit from exercise training by a reduction in fat gain^12,13^ and insulin resistance in skeletal muscle^14^. Endurance exercise training has also been reported to prevent liver fat accumulation in rats^15^ as well as the upregulation of several genes involved in *de novo* lipogenesis^16^. There is also evidence that resistance or strength training in rats, as an alternative to endurance training, prevents or decreases liver fat content^17,18^. Furthermore, RT has been reported to promote muscle strength and hypertrophy in Ovx animals, thus providing protection against menopause-associated sarcopenia and osteopenia^8,17,18^. More recently, it was reported that resistance training restored the gene expression of key molecules involved in *de novo* lipogenesis in livers of ovariectomized rats^8^. We, therefore, used a resistance training program, which can be considered a model of strength training, to test the hypothesis that resistance training ameliorates the increase in GLUT2 expression in livers of Ovx rats. 

## METHODS

### Animal care

Female Holstman rats (*n* = 40) from the animal facility of the University of São Paulo State (UNESP, Araraquara, Brazil) weighting ~ 220 g upon arrival were housed in collective cages. The animals had ad libitum access to food and tap water. All animals were fed with commercial rodent chow. Their environment was controlled in a reverse light cycle (12 h dark starting at 08:00 AM). Food intake was monitored daily over the entire experimental period. Body mass was measured 3 times/week at the same time of day. All experiments described in the present report were conducted according to the Guide for Care and Use of Laboratory Animals^19^ and approved by the Ethics Committee on Animal Use from the Federal University of São Carlos (CEUA-UFSCar) Protocol n° 005/2013.

### Experimental groups

Rats were randomly distributed into five experimental groups (*n* = 8/group). Rats first underwent a bilateral ovariectomy (*n* = 24) or a bilateral sham operation (Sham, *n* = 16). Thereafter, one group of Ovx and one group of Sham rats remained sedentary while one group of Ovx and one group of Sham rats were submitted to a 12-week resistance training program. The third group of Ovx rats remained sedentary and were given 17β-estradiol supplementation. Altogether, five groups were compared: Sham-sedentary (Sed), Sham-resistance trained (RT), Ovx-Sed, Ovx-RT, and Ovx-Sed with hormone replacement (Ovx-E2).

### Surgery

Ovariectomy and sham surgeries were performed when the rats reached 250 g of body mass, according to the technique described by Kalu^20^. To perform this procedure, rats were first anesthetized with a mixture of ketamine-zylazine (61.5 - 7.6 mg/kg, ip). A bilateral incision (1.0 - 1.5 cm) was made through the skin and muscle layers. The ovaries were removed and the skin and muscles were sutured. The sham surgery was performed using the same procedure but the ovaries were not removed. The antibiotic tramadol hydrochloride (20 mg/kg, sc) was injected every 24 h for five days after surgery. The rats were allowed to recover from surgery for seven days, which also permitted us to ensure the systemic effects of Ovx. At the end of this period, rats in the Ovx-E2 group underwent a second surgical procedure to insert silastic capsules under the skin of the neck to provide hormonal replacement.

### Estrogen replacement (E2)

Silastic capsules (15 mm) with internal and external diameter of 1.02 mm and 2.16 mm, respectively (Dow Corning VWR International, Buffalo Grove, IL, USA) were used for E2 replacement. Sunflower oil was used as the vehicle, with a 5% concentration of 17β-estradiol (50 mg/ml oil). Ten μL of this solution was pipetted into each capsule and then both sides were sealed with silastic glue. The capsules were stored for 24 h to allow the glue to dry. After drying, the capsules were kept in saline (0.9%) solution^21^.

### Resistance training protocol

The training sessions consisted of climbing a vertical wooden and metal ladder with the following dimensions: 80° of inclination, 110 cm height x 18 cm width, and 2 cm spacing between the ladder rungs. At the top of the ladder, the animals reached a 20 x 20 x 20 cm cage, which allowed them to rest between climbing sessions. The first three training sessions were used to allow the animal to adapt to the process of climbing and to determine the maximum possible weight load. The load consisted of fishing sinkers that were previously weighed, labeled, and stored in falcon tubes. The falcon tubes were tied to proximal portion of the rats’ tails. The present RT protocol is an adaptation of the program described by Hornberg & Farrar^22^.

### Familiarization step

The animals were first familiarized with the training procedures over two consecutive days. The overload apparatus was attached to the rat’s tail without any weight. The rats were placed at the bottom of the ladder and familiarized with the climb. Once they reached the cage at the top of the ladder, the rats were allowed a 2 min rest period. If necessary, a physical stimulus with fingers or tweezers was applied to the animal’s tail to initiate climbing movements. The rats were considered to have adapted to the ladder protocol when voluntary climbing was performed three consecutive times without any stimulus.

### Maximal load determination step

The maximal load determination was initiated with a load equivalent to 75% of the rat’s body mass. After each successful climb, 30 g were added to the apparatus until the rat was unable to climb the entire length of the ladder. The highest carried load was considered the maximum carrying capacity of each rat.

### Resistance training program

The RT program consisted of training sessions held every 3 days for 12 weeks, for a total of 27 sessions. The training sessions consisted of 4 to 9 climbs with progressively increasing weights. The size of the ladder required the animals to perform 8 - 12 movements per climb. The first four climbs were carried out with loads corresponding to 50, 75, 90, and 100% of the rat’s maximal carrying capacity as determined in the last training session. For the 5 subsequent climbs, an additional 30 g load were progressively added until the rat was unable to climb the entire ladder. The new maximal load determined during the training program was used for the next session.

### Blood and tissue sampling

Rats were euthanized between 9:00 AM and 12:00 PM after removal of food at 7:00 AM. Rats in the trained groups were euthanized 48 h following the last training session. Immediately after decapitation, blood samples were collected and centrifuged at 3000 rpm for 10 minutes at 4°C, and then stored at -20°C. The liver was weighed and then washed with saline (0.9%). The median lobe was used for all molecular analyses.

### Biochemical analyses

Measurement of 17β-estradiol plasma levels was performed by the ELISA technique^23^ using commercial kits (ADI-900-174, Enzo Life Sciences, Farmingdale, NY, USA) according to the manufacturer’s instructions. Liver triglyceride concentrations were determined from glycerol released after KOH hydrolysis according to the technique described by Frayn and Maycock^24^. Liver glycogen content was determined using phenol-sulfuric acid reactions as described by Lo et al.^25^.

### Quantitative real time polymerase chain reaction (RT-PCR)

Total RNA was extracted from frozen livers with the use of the RNA extraction Mini kit (Invitrogen) according to the manufacturer’s protocol. Then, the RNA was treated with DNase (Invitrogen) in order to avoid genomic contamination. Total RNA (2 pg) was reverse-transcribed into complementary DNA (cDNA) using high-capacity cDNA reverse transcription kits (Applied Biosystems). Reverse-transcribed samples were stored at -20°C. GAPDH gene expression was determined using a pre-validated Taqman Gene Expression Assay (Applied Biosystems, Rn01462d61, Foster City, CA). The gene expression levels of target genes were determined using assays designed with the Universal Probe Library from Roche. The primer sets and UPL probe numbers are presented in Table 1. To validate the efficiency of the qPCR assays, we used a mix of the samples tested in the study. An ABI PRISM® 7900HT (Applied Biosystems) was used to detect amplification level and was programmed with an initial step of 3 min at 95°C, followed by 40 cycles for 5 s at 95°C and 30 s at 60°C. All reactions were run in triplicate and the average values of threshold cycle (*C_T_*) were used for quantification. *GAPDH* was used as endogenous control. The relative quantification of target genes was determined using the ΔΔC_T_ method^26^. Briefly, the C_T_ values of target genes were normalized to an endogenous control gene (*GAPDH*) (ΔC_T_ = C_T__Target_ - C_T__GAPDH_) and compared with a calibrator: (ΔΔC_T_ = ΔC_T__Sample_ - ΔC_T__Calibrator_). Relative expression (RQ) was calculated using the Sequence Detection System (SDS) 2.2.2 software (Applied Biosystems) with the formula RQ = 2^-ΔΔC_T_^.

**Table 1.** Oligonucleotide primers used for PRC-RT

Genes	Accession N"	Sence primer(5’-3’)	Antisense primer(5’-3’)
GLUT2	NM_012879.2	CTGGGTCTGCAATTTCATCA	CGTAAGGCCCGAGGAAGT
PEPCK	NM_198780.3	GATGACATTGCCTGGATGAA	AACCGTTTTCTGGGTTGATG
PPARγ	NM_013124.3	TTTATAGCTGTCATTATTCTCAGTGGA	CGGGTGGTTCAGCTTCAG
GAPDH	NM_017008.3	CCCTCAAGATTGTCAGCAATG	AGTTGTCATGGATGACCTTGG

### Protein assay

Protein analyses were performed by the Western blotting method, described by Kurien and Scofield^27^. Aliquots of hepatic lysate (~ 25 mg) were separated by SDS- PAGE gel and transferred to a nitrocellulose membrane. Before the procedure, the membrane was blocked with 5% albumin solution. After this, each membrane was incubated overnight at 4°C with appropriate dilutions of the primary antibodies, including GLUT2 (Sigma-aldrich) and β-actin (Cell Signaling Technology). Each membrane was washed in TBST (3 x 5 min) and then incubated with the appropriate secondary antibody conjugated to HRP for 120 min at room temperature. Antibody binding was detected by enhanced SuperSignal® West Pico Chemiluminescent Substrate (PIERCE, IL, USA), as described by the manufacturer. Blots were scanned and the densitometry of protein bands was determined by pixel intensity using Molecular Imager® ChemiDoc™ XRS+ (Bio-Rad Laboratories Inc., CA, USA) with Image Lab™ 2.0 Software.

### Statistical analysis

Values are expressed as means ± standard error (SE). The data were first analyzed by the Kolmogorov-Smirnov normality test (SPSS 22.1 software) and all results were considered parametric. The one-way analysis of variance (ANOVA) test was used to quantify the RT or E2 variables. The Tukey *post hoc* test was used in the event of a significant (*P* < 0.05) F ratio. 

## RESULTS

Body mass was significantly (*P* < 0.01) higher in Ovx than in Sham rats (Table 2). Exercise training did not significantly affect these measurements, though it did significantly (*P* < 0.01) reduce body mass in Ovx-E2 rats as compared to Sham-Sed animals. On the other hand, food intake was not affected by any of the experimental conditions, while the only change in liver mass was an increase (*P* < 0.01) seen in Ovx-E2 rats. As expected, uterus weight and 17β-estradiol plasma levels were lower (*P* < 0.01) in Ovx compared to Sham animals and were increased (*P* < 0.01) in Ovx-E2 rats (Table 2). There was no significant difference between the Sham and the Ovx rats in their capacity to carrying increasing loads throughout the 12-week resistance training program. The loads carried by both groups of rats increased significantly between the 1st and the 15th session (*P* < 0.01), and again between the 15th and the 27th session (*P* < 0.05). On the whole, carried loads increased from ~ 362 g to ~ 985 g at the end of the 12-week period, thus indicating the efficiency of the resistance training program.

**Table 2.** Anthropometric parameters and food intake

Parameters	Sham-Sed	Sham-RT	Ovx-Sed	Ovx-RT	Ovx-E2
Body mass (g)	308.5 ± 10	315.7 ± 6	358.4 ± 10 ^aa^	339.4 ± 10 ^a^	310.5 ± 11^cc^
Food intakte (g/day)	21.2 ± 0.4	23.1 ± 0.8	23.7 ± 0.9	23.6 ± 0.8	23.8 ± 0.9
Liver mass (g)	8.4 ± 0.2	8.4 ± 0.3	8.3 ± 0.5	8.1 ± 0.3	10.2 ± 0.4^aa^ ^cc^
Uterus mass (g)	0.65 ± 0.07	0.58 ± 0.06	0.09 ± 0.01^aa^	0.10 ± 0.01^aa^	0.59 ± 0.07^cc^
17β-estradiol (pg/ml)	34.0 ± 0.1	34.1 ± 0.1	16.8 ± 0.2 ^aa^	17.2 ± 0.3 ^aa^	44.2 ± 0.9 ^aa^ ^cc^

Values are mean ± SE in Sham and ovariectomized (Ovx) rats in the sedentary (Sed) and resistance training (RT) and in Ovx-Sed rats given 17β-estradiol (E2)

n = 8 rats per group. For 17β-estradiol, n = 4 rats per group

a: Significantly different from Sham-SED (P<0.05)

aa (P<0.01)

c: Significantly different from Ovx-SED (P<0.05)

cc (P<0.01)

**Figure 1.**
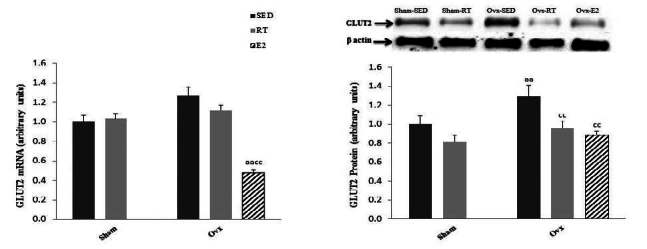
Hepatic mRNA and protein levels of the GLUT2 glucose transporter in Sham and ovariectomized (Ovx) rats in the sedentary (Sed) and resistance training (RT) groups and in Ovx-Sed rats given 17β-estradiol (E2). Values are mean ± SE with n = 8 rats per group for mRNA expression and n = 4 per group for protein expression. a (*P* < 0.05) Significantly different from Sham-SED, aa (P < 0.01), c (*P* < 0.05) Significantly different from Ovx-SED, cc (*P* < 0.01)

Hepatic GLUT2 transcript levels were not significantly affected either by Ovx or training state but were significantly (*P* < 0.01) lower in Ovx animals supplemented with estrogen (Fig. 1). On the other hand, protein levels of GLUT2 in the liver were significantly (*P* < 0.01) higher in Ovx compared to Sed animals, and the levels were back to normal in Ovx-RT and Ovx-E2 rats.

**Figure 2.**
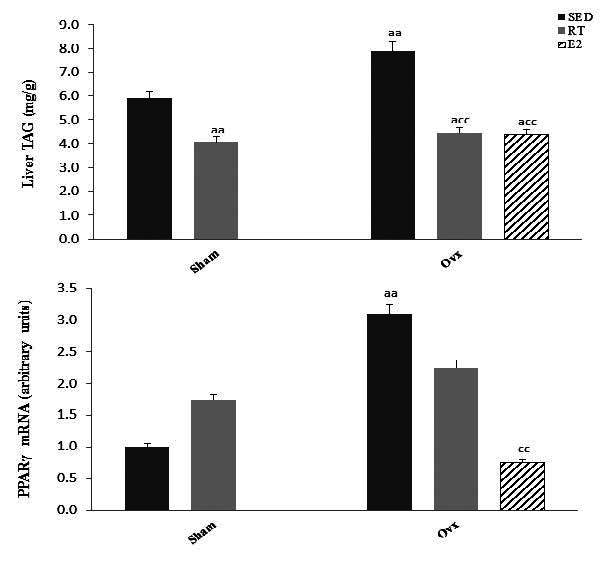
Liver triacylglycerol and hepatic mRNA expression of peroxysome proliferator-activated receptor γ (PPARγ) in Sham and ovariectomized (Ovx) rats in the sedentary (Sed) and resistance training (RT) and in Ovx-Sed rats given 17β-estradiol (E2). Values are mean ± SE with n = 8 rats per group. a (*P* < 0.05) Significantly different from Sham-SED, aa (*P* < 0.01), c (*P* < 0.05) Significantly different from Ovx-SED, cc (*P* < 0.01)

Ovariectomy in the Sed state was associated with a significant (*P* < 0.01) increase in liver fat (Fig. 2). Exercise training, however, significantly (*P* < 0.01) lowered hepatic fat content in Ovx as well as in Sham rats. E2 replacement reduced (*P* < 0.05) hepatic fat content to levels even under those of Sham-Sed rats. As for GLUT2, gene expression of the transcription factor PPARγ was significantly (*P* < 0.01) higher in Ovx-Sed compared to Sham-Sed rats (Fig. 1). This difference was not observed in Ovx-RT animals, indicating that training reduced gene expression of PPARγ in Ovx rats. E2 replacement in Ovx rats significantly (*P* < 0.01) lowered PPARγ transcript levels in Ovx animals.

**Figure 3.**
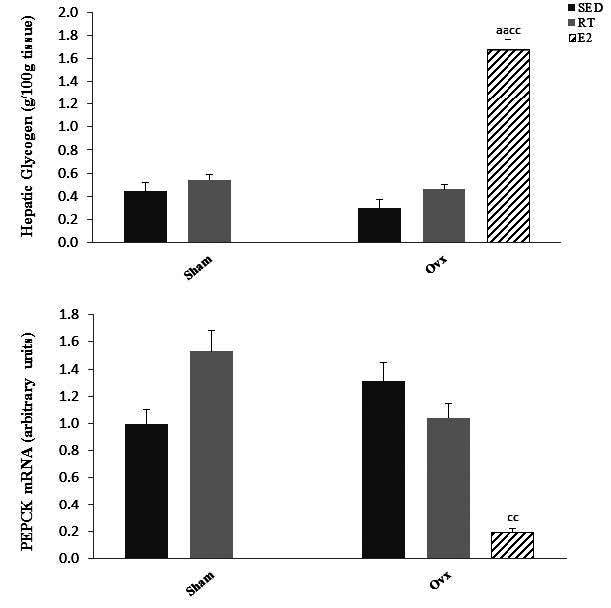
Glycogen levels and mRNA expression of phosphoenolpyruvate carboxykinase (PEPCK) in Sham and ovariectomized (Ovx) rats in the sedentary (Sed) and resistance training (RT) and in Ovx-Sed rats treated with 17β-estradiol (E2). Values are mean ± SE with n = 8 rats per group. a (*P* < 0.05) Significantly different from Sham-SED, aa (*P* < 0.01) c (*P* < 0.05). Significantly different from Ovx-SED, cc (*P* < 0.01)

To gain insight into liver glucose metabolism, we measured liver glycogen concentration and gene expression of phosphoenolpyruvate carboxykinase (PEPCK), the main enzyme in the gluconeogenesis pathway. Hepatic glycogen levels were not affected by ovariectomy or training state but were highly (*P* < 0.01) increased by E2 replacement (Fig. 3). Gene expression of PEPCK was not affected by ovariectomy or training state but was significantly (*P* < 0.01) lower in Ovx-E2 compared to Ovx- Sed rats (Fig. 3). 

## DISCUSSION

### Ovariectomy

The results of the present study first indicated that the well-documented fat accumulation in livers of Ovx rats is accompanied by an increase in the expression of GLUT2, the major glucose transporter in the plasma membranes of hepatocytes, along with an increase in gene expression of the transcription factor PPARγ, which is known to modulate GLUT2 gene expression^11^. These results suggest that alterations in liver fat metabolism, known to ocur in Ovx rats, such as increased *de novo* lipogenesis, also affect hepatic glucose uptake. In addition, the present data indicate that these disturbances in liver fat and glucose transport may be overcome by a regular resistance training program. These results reinforce the concept that exercise training may alleviate some of the metabolic consequences of low estrogenic status.

Liver fat content was increased by about 35% in Ovx rats in this study. Our hypothesis that Ovx in rats may lead to an increase in gene expression of the GLUT2 transporter was based on previous findings that fat accumulation in the livers of Ovx animals is associated with an increase in *de novo* lipogenesis^7,8^. Increased lipogenesis logically requires more glucose molecules to be available as substrates, thus, hypothetically, leading to increased glucose uptake through GLUT2 transport. Studies in GLUT2 knockout mice confirm that, indeed, glucose uptake by hepatocytes is a major substrate for lipogenesis^28^. Although the basic function of GLUT2 is to catalyze the passive transport of glucose across the plasma membrane, it is required for glucose uptake, whereas it is dispensable for glucose output^29^. Also supporting the link between liver fat accumulation and GLUT2 transport is the finding that glucose-induced SREBP- 1c upregulates GLUT2 mRNA in primary hepatocytes of mouse liver^30,31^. SREBP-1c is a well-documented regulator of lipogenesis and has been repeatedly reported to be upregulated in Ovx animals^8,32^. Altogether, the present data are in line with the concept that an increase in fat content in livers of Ovx rats is linked to increased glucose uptake through an increased expression of GLUT2 protein.

The increase in gene expression of the PPARγ transcription factor supports the increase in GLUT2 synthesis in the livers of Ovx rats. PPARγ expression is low in liver tissue^10^. Nevertheless, it has been reported that PPARγ is able to modulate gene expression of GLUT2 in liver^11^. Ankaflavin, a natural PPARγ agonist, has been shown to enhance insulin sensitivity by increasing hepatic GLUT2 expression and glucose uptake in rats^33^. There is little doubt that the increase in fat content in the liver cells of Ovx animals is related to a reduction in plasma estradiol levels^18,34^. The present data, therefore, support the existence of a link between liver fat accumulation in Ovx rats at least partially through increased *de novo* lipogenesis and GLUT2 transport and an increase in PPARγ gene expression.

From a mechanical point of view, it is possible that the present increase in GLUT2 gene expression in Ovx animals is linked to a transient decrease in intracellular glucose level secondary to increased lipogenesis. Glucose has been reported to induce a dose-dependent increase in GLUT2 mRNA levels in cultured hepatocytes, while insulin had no effects^35^. To go one step further into hepatic glucose metabolism in Ovx animals, we measured glycogen levels and PEPCK mRNA levels. Both of these parameters remain unchanged following estrogen withdrawal. The absence of an effect on glycogen levels in Ovx rats indicate that if indeed glucose uptake is increased in the present Ovx rats, it was not used for glycogen synthesis. Actually, an increase in liver fat, as seen in the present Ovx rats, is associated with a reduction in glycogen storage^36^. There is also no indication that gluconeogenesis is increased for estrogen withdrawal, indicating no shortage of glucose.

### Estrogen Replacement

E2 replacement in Ovx rats resulted, as would be expected, in a reduction in liver fat levels but also in a large increase in liver glycogen levels and a reduction in gene expression of PEPCK. The 5% hormone concentration used in the present study appears to be an effective replacement over a three-week period^21^. However, the present replacement over a 12-weeks period has led to supraphysiological plasma levels of estrogen, as judged from the high estrogen plasma concentrations and the increase in liver weight. Nevertheless, the present E2 replacement data are in line with a previous report indicating that treatment with estradiol in Ovx mice increases liver glycogen and reduces gluconeogenesis^37^.

### Resistance training

Training has been reported to be effective in reducing liver fat accumulation in Ovx animals^17,18,38^. The present study extended these findings by showing that GLUT2 protein and PPARγ transcript levels were restored almost to normal levels in the livers of Ovx rats submitted to resistance training. This at first glance supports the link between liver fat accumulation through increased *de novo* lipogenesis and GLUT2 transporters. The present RT program is an interesting alternative to classical endurance training. Similar RT programs have been used successfully in previous studies, leading to similar reductions in liver fat accumulation^17,38,39^. It is intriguing that RT and E2 replacement in the present study led to similar metabolic improvements in the livers of Ovx animals. The same phenomenon has been previously observed on the reduction of subclinical inflammation in Ovx rats^16^. The present data are, therefore, in line with the concept that exercise training has estrogenic-like effects.

## CONCLUSION

The present study indicates that estrogen withdrawal-induced liver GLUT2 protein and PPARγ mRNA in Ovx rats are accompanied by an increase in fat accumulation, and that both of these responses are normalized when rats are submitted to a resistance training program. These results suggest that, in addition to fat metabolism, the level of estrogens also affects hepatic glucose metabolism. From a clinical point of view, the present data reiterate the importance of exercise training to alleviate some of the metabolic consequences of low estrogenic status in postmenopausal women. In line with this idea, it is interesting to note the recent finding that rats selectively bred for high intrinsic aerobic fitness are protected from ovariectomy-induced metabolic disorders^40^.
